# Use of a novel integrated dilator-needle system in cryoballoon procedures: a zero-exchange approach

**DOI:** 10.1007/s10840-022-01294-x

**Published:** 2022-07-07

**Authors:** Sing-Chien Yap, Rohit E. Bhagwandien, Tamas Szili-Torok

**Affiliations:** Department of Cardiology, Erasmus MC, University Medical Center Rotterdam, 3015 GD Rotterdam, The Netherlands

**Keywords:** Air embolism, Atrial fibrillation, Cryoballoon, Pulmonary vein isolation, Transseptal needle, Transseptal puncture

## Abstract

**Background:**

Recently, a novel integrated dilator-needle system (AcQCross Qx, Acutus Medical) was introduced to reduce the number of exchanges for a transseptal access. This system can be used in combination with large bore sheaths. In this pilot study, we evaluated the safety and efficacy of a zero-exchange approach with the AcQCross system in cryoballoon procedures.

**Methods:**

In this retrospective single-center study, we included 40 patients (AcQCross: *n* = 20; control group: *n* = 20) who underwent a cryoballoon procedure for the treatment of atrial fibrillation. In the AcQCross and control group, patients underwent ablation with POLARx (Boston Scientific) and Arctic Front Advance Pro (AFA-Pro, Medtronic) in equal numbers (*n* = 10). In the AcQCross group, the AcQGuide Max sheath (Acutus Medical) was used in all POLARx cases.

**Results:**

The baseline characteristics of the study population were comparable between groups. In the AcQCross group, there was a reduction in procedure time (49.7 ± 9.0 min vs. 59.6 ± 8.1 min, *P* < 0.001) and time from puncture until balloon delivery (15.5 ± 6.8 min vs. 21.5 ± 7.4 min, *P* = 0.01) in comparison with the control group. The balloon in body time, fluoroscopy time, number of cryoapplications, and biophysical parameters were similar between groups. There was one temporary phrenic nerve injury in the AcQCross group. Importantly, no signs of air embolism were noted with the AcQGuide Max sheath.

**Conclusions:**

The use of the novel AcQCross system improves procedural efficacy in cryoballoon procedures by reducing the number of exchanges.

## Introduction

Cryoballoon ablation (CBA) has become a well-established approach to achieve pulmonary vein isolation (PVI) for the treatment of atrial fibrillation (AF) [1]. The major advantages are the shorter procedure duration, lower interoperator variability, shorter learning curve, and safety in comparison with radiofrequency (RF) ablation [1–4]. Over the years, the approach to PVI with a cryoballoon has undergone refinements and modifications to optimize the procedural workflow [5]. These measures include the following: single-freeze strategy (eliminating a bonus freeze), shorter freezing application (180 versus 240 s), time-to-isolation (TTI)-guided ablation, shorter distal tip, radiation reduction, and the use of a figure-of-8 suture [6–12].

During cryoballoon procedures, the transseptal access is usually gained through a standard 8F transseptal sheath with the use of a transseptal needle. After placement of the guidewire in the pulmonary vein (PV), the transseptal sheath is replaced by a large bore sheath (15.0 to 15.9F) to accommodate the cryoballoon. Recently, a novel integrated dilator-needle system (AcQCross Qx, Acutus Medical, Carlsbad, CA) became available (US FDA clearance in 2021 and CE mark in 2020). In the AcQCross system, the dilator and transseptal needle form a single component. The lumen of the tapered-tip shaft is fitted with a hollow stainless steel transseptal needle, and both the shaft and needle are connected to the same proximal handle. The AcQCross system allows a 0.032″ guidewire to be loaded during the transseptal puncture (TSP) [13]. This provides the ability to position, reposition, and cross the fossa ovalis without removing the guidewire. The hollow needle is affixed to a spring-tensioned actuator that prevents needle extension until the operator purposely advances the needle via a slider button located on the proximal handle. The AcQCross family of sheaths has specific integrated dilator-needle systems for large bore sheaths such as the 15.2F AcQGuide Max 2.0 steerable sheath (Acutus Medical) and the 15.0F FlexCath Advance sheath (Medtronic, Minneapolis, MN). Thus, the AcQCross system streamlines the transseptal procedural workflow in cryoballoon procedures by eliminating the need for transseptal sheath exchange.

### Aim and hypothesis of the study

The aim of the present pilot study was to evaluate the safety and efficacy of a zero-exchange approach with the AcQCross system in cryoballoon procedures. We hypothesize that the use of AcQCross is safe and results in reduction in the procedure time by a more efficient delivery of the cryoballoon in the left atrium (LA).

## Methods

### Study population

In this retrospective nonrandomized single-center study, we included 40 patients who underwent a first‐time CBA for the treatment of symptomatic paroxysmal or persistent AF. The AcQCross group consisted of the first 10 consecutive patients who underwent ablation with AcQCross and POLARx cryoablation catheter (Boston Scientific, Marlborough, MA) and the first 10 consecutive patients who underwent ablation with AcQCross and Arctic Front Advance Pro cryoablation catheter (AFA-Pro, Medtronic). The historical control group consisted of the last 10 consecutive patients undergoing ablation with POLARx and the last 10 consecutive patients undergoing ablation with AFA-Pro before the introduction of AcQCross in our clinical practice.

### Periprocedural management

All patients received oral anticoagulation for at least 3 weeks before ablation. Direct-acting oral anticoagulants were withheld in the morning of the procedure. Vitamin K antagonists continued with a target INR between 2.0 and 2.5. To exclude left atrial thrombi, all patients underwent transesophageal echocardiogram just before the procedure.

### Transseptal access

**Fig. 1:**
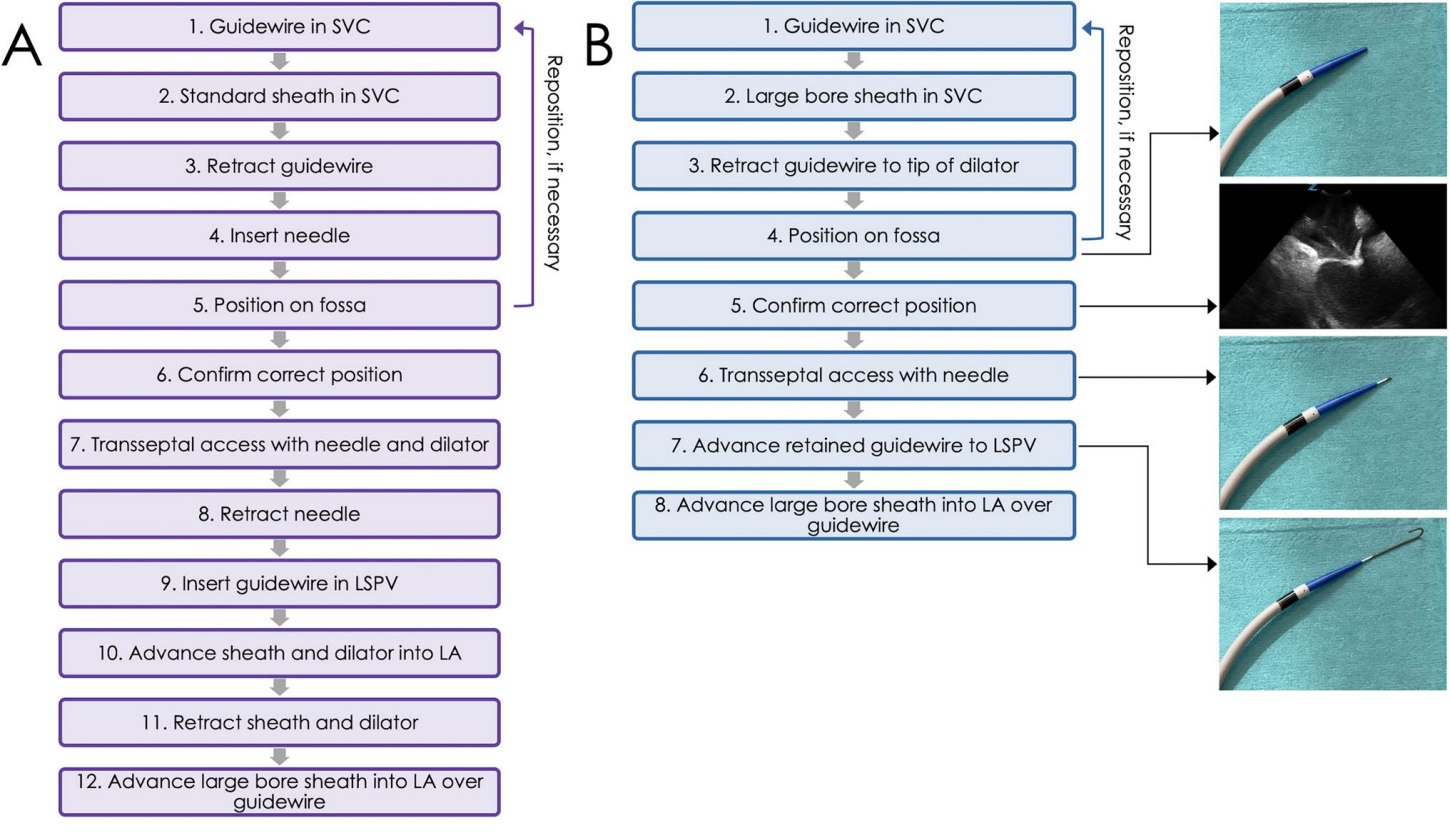
Overview of the transseptal procedural workflow with large bore sheaths. **A** Conventional transseptal workflow. **B** Transseptal workflow with the AcQCross system. The photos depict a AcQCross system in a AcQGuide Max 2.0 sheath. The intracardiac echocardiographic image shows tenting of the fossa ovalis during correct positioning of the dilator-sheath combination. LA, left atrium; LSPV, left superior pulmonary vein; SVC, superior vena cava

All procedures were performed under deep sedation by two experienced cryoballoon operators (SCY, REB). Femoral vein punctures were performed under ultrasound guidance. After placement of 2 short introducer sheaths (8F and 10F) in the femoral vein, a bolus of intravenous heparin (5000 IE) was given. In the control group, a 0.032″ guidewire was placed from the femoral access site to the superior vena cava (SVC) (Fig. 1A). The short introducer sheath was removed, and a SL1 sheath (Swartz, Abbott, Abbott Park, IL) was placed over the guidewire to the SVC. The guidewire was replaced by a RF transseptal needle (NRG, Bayliss Medical, Rouyn-Noranda, Canada). After a smooth drag-down maneuver, the tip of the SL1 sheath was placed on the fossa ovalis guided by intracardiac imaging (ICE) (ViewFlex Xtra, Abbott). A TSP was performed with the RF transseptal needle. After puncture of the fossa ovalis with the needle and advancement of the dilator across the fossa ovalis, the transseptal needle was replaced by a guidewire which was placed in the left superior PV (LSPV). Finally, the SL1 sheath was replaced by a large bore steerable sheath (either POLARSHEATH [Boston Scientific] or FlexCath Advance) into the LA. The dilator and guidewire are slowly pulled out, and the sheath is flushed thoroughly before introduction of the cryoballoon catheter.

In the AcQCross group, a 0.032″ guidewire was placed from the femoral access site to the SVC (Fig. 1B). After removal of the short introducer sheath, the femoral access site was predilated with the dilator of the 10F sheath. Thereafter, the combination of the AcQCross system and the large bore steerable sheath (either FlexCath Advance or AcQGuide Max 2.0) was placed over the guidewire to the SVC. The guidewire was retracted to the tip of the dilator, thus retained in the dilator, and after a drag-down, the tip of the dilator was placed on the fossa ovalis as guided by ICE. By advancing the slider button forward on the AcQCross proximal handle, a hollow stainless steel needle protrudes from the dilator and punctures the fossa ovalis (Fig. 1B). It is also possible to deliver RF on the needle in case of a thickened fossa ovalis. In our case series, we did not need RF to cross the fossa ovalis. Directly after the puncture, the guidewire can be advanced to the LSPV after which the large bore sheath can cross the septum. Then, the dilator and guidewire are slowly pulled out, and the sheath is flushed thoroughly before introduction of the cryoballoon catheter. After achieving transseptal access, intravenous heparin was administered to achieve a target-activated clotting time of ≥ 300 s.

### Ablation procedure

Patients underwent PVI using either the POLARx or AFA-Pro cryoablation catheter. Because there is no dedicated AcQCross system for the POLARSHEATH, we used the 15.2F AcQGuide Max sheath in all 10 patients in the AcQCross group who underwent ablation with a POLARx cryoballoon. We deemed the FlexCath Advance sheath too small for the POLARx cryoballoon. In patients undergoing ablation with AFA-Pro in the AcQCross group, both the AcQGuide Max (*n* = 4) and FlexCath Advance steerable sheaths (*n* = 6) were used. After optimal PV occlusion was achieved, as assessed by contrast injection, cryoablation was started. A TTI-guided ablation protocol was used. The freeze duration was 180 s if TTI was < 60 s; otherwise, a 240‐s freeze cycle was employed. No bonus freeze was employed routinely. PVI was confirmed by entrance/exit block at the end of the procedure. During cryoablation of the right‐sided PVs, high‐output right phrenic nerve stimulation was performed using a diagnostic catheter in the right subclavian vein or superior vena cava. Diaphragmatic excursion was assessed by palpation or, in case of the POLARx system, by using the diaphragmatic movement sensor (DMS). The DMS uses an accelerometer and provides a relative measure of the diaphragmatic excursion. Whenever the diaphragmatic excursions decreased or the DMS percentage drops below a cutoff (65%), cryoablation was immediately terminated. During cryoablation of the left‐sided PVs, a diagnostic catheter was placed in the right ventricle to provide ventricular pacing in case of a vagal response after cryoablation. The day following the procedure, the groin was inspected for groin hematoma, and a transthoracic echocardiogram was performed to rule out pericardial effusion.

### Data collection and study endpoints

Patient demographic and clinical data were obtained from the medical records. For every CBA, the following parameters were collected: grade of PV occlusion (semiquantitative grades 1 to 4), duration of CBA, and TTI (if measurable). For CBA applications > 120 s, we collected the balloon nadir temperature and thawing time until 0 °C.

The primary efficacy endpoints were procedure time (defined as time from puncture until removal of the last sheath), time from puncture until balloon delivery, balloon in body time, and fluoroscopy time. The primary safety endpoint was defined as a composite of stroke, transient ischemic attack (TIA), air embolism (including transient ST elevation), cardiac tamponade, and myocardial infarction.

### Statistical method

Continuous data are presented as mean ± standard deviation (SD) or median with 25th and 75th percentile, as appropriate. Categorical variables are presented by frequencies and percentages. Differences between continuous variables were tested with the Student’s *t*-test and Mann–Whitney *U*‐test. Differences between categorical variables were evaluated using the chi-square test or Fisher’s exact test in case of low numbers per cell. Statistical analyses were performed using SPSS (version 28.0.1.0). *P*-values < 0.05 were considered statistically significant.

## Results

### Study population

In total, 40 patients were included in this study. In both groups, there were 10 patients treated with POLARx and 10 patients with AFA-Pro. The baseline characteristics between the AcQCross group and control group were similar (Table 1).

**Table 1:** Baseline characteristics

	AcQCross group (*n* = 20)	Control group (*n* = 20)	*p*-value
Age, years	61.2 ± 11.6	61.8 ± 7.1	0.83
Male sex	15 (75%)	11 (55%)	0.19
BMI (kg/m^2^)	27.9 ± 4.9	27.2 ± 4.4	0.65
Hypertension	6 (30%)	5 (25%)	0.72
Diabetes mellitus	1 (5%)	0 (0%)	1.00
Previous stroke/TIA	0 (0%)	1 (5%)	1.00
OSAS	2 (10%)	1 (5%)	1.00
Coronary artery disease	2 (10%)	2 (10%)	1.00
Paroxysmal AF	15 (75%)	15 (75%)	1.00
Persistent AF	5 (25%)	5 (25%)	1.00
LA dimension (mm)	39.5 ± 7.3	36.6 ± 4.7	0.14
LAVI (mm^2^)	29.9 ± 10.5	30.9 ± 10.5	0.78
LVEF (%)	54.0 ± 9.9	58.4 ± 5.3	0.09
LVEF < 50%	4 (20%)	2 (10%)	0.66
CHA_2_DS_2_-VASc score	1 (0–3)	2 (0–2)	0.90
DOAC	19 (95%)	20 (100%)	1.00
VKA	1 (5%)	0 (0%)	1.00
Antiarrhythmic drug	17 (85%)	18 (90%)	1.00
Flecainide	2 (10%)	5 (25%)	0.41
Beta-blocker	9 (45%)	7 (35%)	0.52
Sotalol	7 (35%)	5 (25%)	0.49
Amiodarone	4 (20%)	3 (15%)	1.00
Digoxin	2 (10%)	0 (0%)	0.49
Verapamil	0 (0%)	3 (10%)	0.23

Continuous data are presented as mean ± SD or median (IQR). Categorical data are presented as *n* (%). *AF*, atrial fibrillation; *BMI*, body mass index; *DOAC*, direct-acting oral anticoagulant; *LA*, left atrium; *LAVI*, left atrial volume index; *LVEF*, left ventricular ejection fraction; *OSAS*, obstructive sleep apnea syndrome; *TIA*, transient ischemic attack; *VKA*, vitamin K antagonist

### Procedural efficacy

**Fig. 2:**
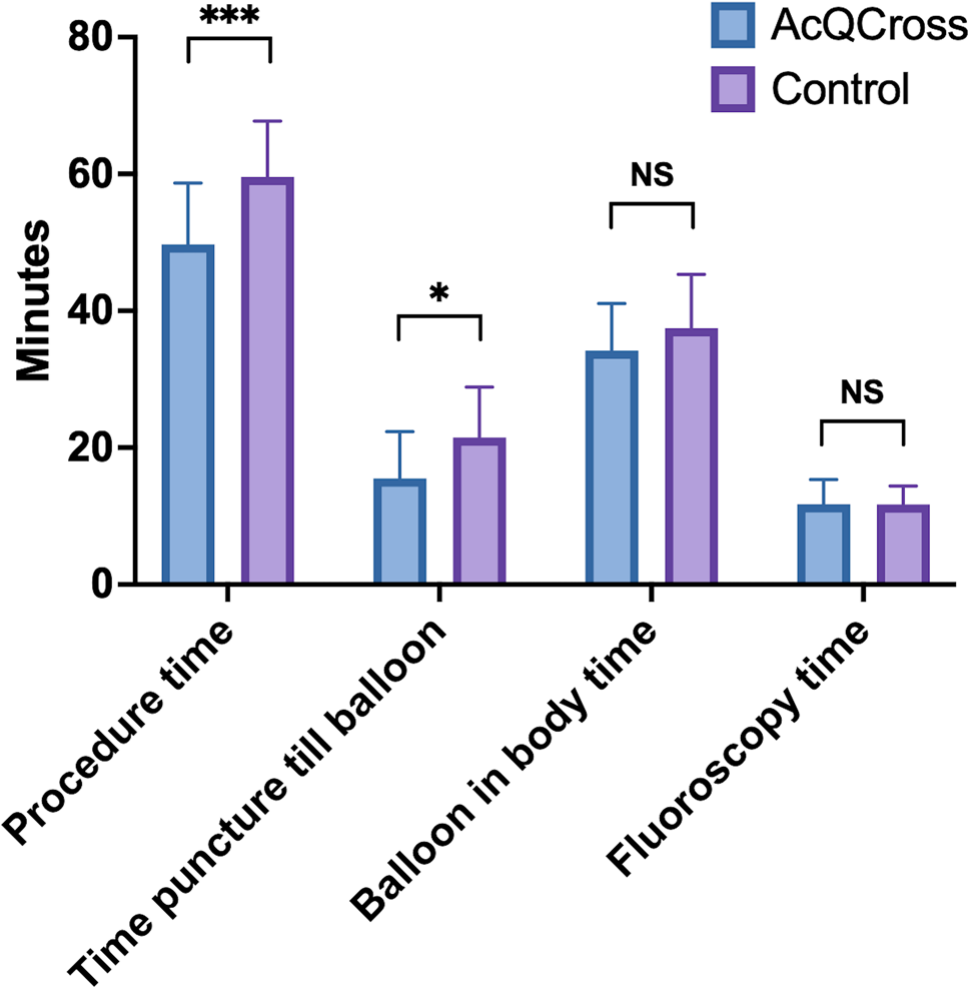
Procedural efficacy between the AcQCross group and the control group. *P < 0.05, ***P < 0.001. *NS* not significant

In the AcQCross group, there was a reduction in procedure time (49.7 ± 9.0 vs. 59.6 ± 8.1, *P* < 0.001) and time from puncture until balloon delivery (15.5 ± 6.8 vs. 21.5 ± 7.4, *P* = 0.01) in comparison with the control group (Table 2) (Fig. 2). The mean difference in time from puncture until balloon delivery was 6.0 min (95% confidence interval, 1.5–10.5 min). The balloon in body time, fluoroscopy time, and the number of cryoapplications were similar between groups. No differences in biophysical parameters and number of cryoapplications per PV were demonstrated between groups (Table 3). The minimal balloon temperature and thawing times are presented separately for POLARx and AFA-Pro because previous studies have demonstrated that these biophysical parameters differ between both systems [14].

**Table 2:** Procedural characteristics

	AcQCross group (*n* = 20)	Control group (*n* = 20)	*p*-value
Procedure time (min)	49.7 ± 9.0	59.6 ± 8.1	< 0.001
Time from puncture until balloon (min)	15.5 ± 6.8	21.5 ± 7.4	0.01
Balloon in body time (min)	34.2 ± 6.9	37.5 ± 7.9	0.17
Fluoroscopy time (min)	11.8 ± 3.6	11.7 ± 2.7	0.96
Total number of CBA	4 (4–6)	4.5 (4–5.5)	1.00

Continuous data are presented as mean ± SD or median (IQR). Categorical data are presented as *n* (%). *CBA*, cryoballoon application

**Table 3:** Procedural and biophysical characteristics for individual PVs

	AcQCross group (*n* = 20)	Control group (*n* = 20)	*p*-value
LSPV	20	19	
Number of CBA	1 (1–1.5)	1 (1–1.5)	0.97
First-freeze isolation	16 (80%)	14 (74%)	0.72
Total freezing duration	210 (180–265)	240 (180–272)	0.90
Minimal temperature, °C	P: − 60 ± 5 A: − 50 ± 6	P: − 62 ± 5 A: − 51 ± 5	0.35 0.57
TTI, s	41 (33–51)	38 (30–60)	1.00
Thawing time to 0 °C, s	P: 21 ± 8 A: 11 ± 3	P: 23 ± 5 A: 15 ± 3	0.44 0.09
LIPV	20	19	
Number of CBA	1 (1–1)	1 (1–1)	0.59
First-freeze isolation	20 (100%)	18 (95%)	0.49
Total freezing duration	180 (180–180)	180 (180–240)	0.25
Minimal temperature, °C	P: − 57 ± 5 A: − 47 ± 3	P: − 57 ± 7 A: − 47 ± 5	0.94 0.82
TTI, s	31 (24–49)	35 (25–71)	0.65
Thawing time to 0 °C, s	P: 20 ± 4 A: 8 ± 1	P: 18 ± 3 A: 9 ± 3	0.42 0.93
LCV	0	1	
Number of CBA		1	-
First-freeze isolation		1 (100%)	-
Total freezing duration		180	-
Minimal temperature, °C		P: − 63	-
TTI, s		50	-
Thawing time to 0 °C, s		29	-
RSPV	16	19	
Number of CBA	1 (1–2)	1 (1–1)	0.27
First-freeze isolation	11 (69%)	17 (90%)	0.21
Total freezing duration	199 (180–248)	180 (180–240)	0.23
Minimal temperature, °C	P: − 64 ± 4 A: − 53 ± 8	P: − 64 ± 3 A: − 55 ± 6	0.99 0.42
TTI, s	32 (23–45)	33 (25–54)	0.64
Thawing time to 0 °C, s	P: 24 ± 6 A: 12 ± 6	P: 26 ± 4 A: 18 ± 3	0.63 0.09
RIPV	16	19	
Number of CBA	1 (1–1)	1 (1–1.5)	0.50
First-freeze isolation	14 (88%)	16 (84%)	0.67
Total freezing duration	180 (180–240)	180 (180–246)	0.88
Minimal temperature, °C	P: − 61 ± 3 A: − 47 ± 4	P: − 62 ± 5 A: − 50 ± 6	0.60 0.20
TTI, s	32 (25–45)	34 (26–46)	0.96
Thawing time to 0 °C, s	P: 25 ± 6 A: 8 ± 3	P: 22 ± 3 A: 9 ± 2	0.30 0.56
RCV	4	1	
Number of CBA	2.5 (2–3)	2	1.00
First-freeze isolation	3 (75%)	1 (100%)	1.00
Total freezing duration	411 (372–450)	220	0.40
Minimal temperature, °C	P: − 62 ± 8 A: − 49 ± 8	P: − 58	- -
TTI, s	51 (27–58)	27	0.67

Continuous data are presented as mean ± SD or median (IQR). Categorical data are presented as *n* (%). Biophysical parameters only reported for cryoapplications > 120 s. *A*, Arctic Front Advance Pro; *CBA*, cryoballoon application; *LCV*, left common pulmonary vein; *LIPV*, left inferior pulmonary vein; *LSPV*, left superior pulmonary vein; *P*, POLARx; *RCV*, right common pulmonary vein; *RIPV*, right inferior pulmonary vein; *RSPV*, right superior pulmonary vein; *TTI*, time to isolation

### Procedural safety

There was no occurrence of a primary safety endpoint in either group. In the AcQCross group, there was one phrenic nerve injury in a patient treated with AFA-Pro. This phrenic nerve injury resolved during the procedure.

## Discussion

In the present pilot study, we demonstrate the feasibility of a zero-exchange transseptal access with large bore sheaths for cryoballoon procedures using an over-the-wire technique with the novel AcQCross system. This approach improves procedural efficacy demonstrated by a reduction of time from puncture until balloon delivery. No signs of air emboli, TIA, or stroke were noted during the procedures. To the best of our knowledge, this is the first case series of a zero-exchange approach in CBA procedures using an over-the-wire technique.

The transeptal puncture remains an important source of complications during an electrophysiological procedure. Over the decades, there have been several modifications to improve the efficacy of TSP while retaining a high safety profile. Currently, several electrophysiology procedures require the placement of a large bore sheath into the LA, e.g., cryoballoon procedures and percutaneous left atrial appendage closure. In the conventional workflow (Fig. 1A), several exchanges are necessary before the large bore sheath is finally placed into the LA, and therapy can be delivered. Furthermore, Miyazaki et al. previously demonstrated that silent cerebral events/lesions secondary to air emboli occur in a significant number of CBA procedures [15]. Reduction of the number of exchanges may potentially reduce air emboli.

Recently, the AcQCross Qx system was introduced which is an integrated dilator-needle combination that can be used with many commercial transseptal sheaths. This system allows the retention of a 0.032″ guidewire while performing the TSP. With the AcQCross system, it is possible to place a large bore sheath into the LA without any exchange of guidewires, needles, or sheaths (Fig. 1B). The curved dilator of the AcQCross system makes it possible to correctly position the dilator on the fossa ovalis. There are several potential advantages of a zero-exchange approach: less risk of air emboli and thromboembolism due to reduction of the number of sheath exchanges, enhancement of procedural workflow, and cost reduction due to elimination of an additional transseptal sheath and needle.

In the present study, we evaluated the feasibility of a zero-exchange approach using the AcQCross system in cryoballoon procedures. We demonstrated that the use of the AcQCross system improved procedural efficacy by reducing the time from puncture until balloon delivery. The reduction in time from puncture until balloon delivery can be explained by a reduction in the number of exchanges. Furthermore, the scrub-in nurse can already prepare the cryoablation catheter, while the physician is performing the TSP, because the large bore sheath is already prepped. By reducing the number of exchanges and shortening the preparation time, the time from puncture until balloon delivery can be shortened significantly.

There are specific preparations needed for delivery of the AcQCross system. After introduction of the dilator-needle combination in the large bore sheath during the preparation phase, it is important to check the functionality of the needle protrusion mechanism. Furthermore, we predilate the femoral access to ease the passage of the curved dilator and sheath through the subcutaneous route and femoral vein wall puncture site. This prevents kinking of the guidewire in case of femoral passage with increased resistance. If it is not possible to mechanically puncture the fossa ovalis (not the case in our case series), there is the option to use RF on the needle by connecting the system to a standard electrosurgery pencil. The AcQCross is the only commercially available transseptal access system which is cleared for both mechanical and RF crossing. It is not clear whether RF on a hollow needle may cause electrocautery tissue coring [16]. Alternatively, the curve of the dilator can be modified on the guidewire to have a more perpendicular approach to the fossa ovalis.

Currently, there is a specific AcQCross system for the FlexCath Advance sheath but not for the POLARSHEATH. Interestingly, Medtronic has recently (April 2022) acquired the left-heart access portfolio, including the AcQCross Qx system, from Acutus Medical. We used the AcQGuide Max 2.0 sheath in all POLARx cases and in a few AFA-Pro cases. There are small differences in the specifications of the different sheaths (Table 4). The inner diameter of the AcQGuide Max 2.0 is slightly larger than the FlexCath Advance and slightly smaller than the POLARSHEATH. We previously demonstrated that a POLARx procedure can be performed with the AcQGuide Max 2.0 sheath [13]. The slightly larger inner diameter of the AcQGuide Max sheath in comparison with the FlexCath Advance sheath makes it easier to resheath the AFA-Pro cryoablation catheter. No signs of coronary air emboli were noted when using the AcQGuide Max 2.0 sheath in combination with the POLARx or AFA-Pro cryoballoon. The use of a different sheath for the POLARx cases did not impact the balloon in body time. Although we used the AcQGuide Max 2.0 sheath for POLARx cases, it is important to highlight that the use of a transseptal sheath from a different manufacturer than the cryoballoon has the inherent risk of incompatibility because this specific combination was not tested for use.

**Table 4:** Overview of the different large bore sheaths

	FlexCath Advance (Medtronic)	POLARSHEATH (Boston Scientific)	AcQGuide Max 2.0 (Acutus Medical)
Sheath outer diameter	15.0F	15.9F	15.2F
Sheath inner diameter	12.0F	12.7F	12.4F
Sheath deflection	135°	155°	180°
Overall length	81 cm	82 cm	85 cm
Usable length	65 cm	68 cm	70 cm
Usable dilator length	87 cm	85 cm	87.4 cm
Guidewire compatibility	0.032″ and 0.035″	0.032″ and 0.035″	0.032″ and 0.035″

Besides the AcQCross system, the VersaCross RF system (Baylis Medical) was recently introduced aiming to reduce the number of exchanges during a TSP [17]. This is a pigtail or J-tip wire-based system which is used as a support guidewire and has a RF tip enabling TSP. After confirming correct positioning on the fossa ovalis, RF is delivered to the tip for TSP followed by placement of the guidewire to the LSPV to serve as a support wire. The transseptal sheath is then replaced by a large bore sheath. Demo et al. has recently demonstrated the feasibility and efficacy of this system in CBA procedures [18]. However, it is important to realize that with the VersaCross system, you still need to exchange the standard transseptal sheath for the large bore sheath; thus, it is not a zero-exchange approach like the AcQCross system.

Although we present an over-the-wire TSP technique, Ströker et al. previously demonstrated the safety and efficacy of a direct approach with the FlexCath Advance sheath using an over-the-needle technique [19]. By preshaping the sheath-dilator assembly, they could target the low anterior or medial portion of the fossa ovalis. After TSP with the 89-cm Brockenbrough needle (BRK, Abbott), the dilator and sheath were carefully advanced under transesophageal echocardiographic guidance. A supporting wire was only necessary in 1% of cases despite that 13% had a challenging interatrial anatomy. In a subanalysis of 30 patients in each arm, this approach was associated with a shorter time to LA in comparison with the conventional over-the-wire approach.

## Study limitations

This was a retrospective nonrandomized study with its inherent limitations. However, all study endpoints are collected as part of standard clinical practice. Furthermore, all procedures were performed by experienced operators. We cannot comment on the presence of cerebral micro-embolization because we did not perform intraprocedural transcranial Doppler or post-ablation diffusion-weighted magnetic resonance imaging. It is expected that reduction in sheath exchanges is associated with a reduction of cerebral micro-embolization, but this should be further evaluated in future research. Finally, considering the nonrandomized study design, all results of this study should be interpreted with caution.

## Conclusion

In this pilot study, we demonstrated that the use of the novel AcQCross system improves procedural efficacy in cryoballoon procedures by reducing the time from puncture until balloon delivery. Importantly, there were no signs of coronary air emboli, TIA, or stroke. Larger international registries are necessary to confirm the safety and efficacy of this novel integrated needle-dilator system. We believe that the AcQCross system may be useful tool to optimize the procedural transseptal workflow during a CBA procedure.

## Data Availability

The data that support the findings of this study are available from the corresponding author upon reasonable request.

## Abbreviations

AF: Atrial fibrillation
CBA: Cryoballoon ablation
DMS: Diaphragmatic movement sensor
ICE: Intracardiac echocardiography
LA: Left atrium
LSPV: Left superior pulmonary vein
PV: Pulmonary vein
PVI: Pulmonary vein isolation
RF: Radiofrequency
SVC: Superior vena cava
TIA: Transient ischemic attack
TSP: Transseptal puncture
TTI: Time to isolation

## References

[1] Andrade JG, Wazni OM, Kuniss M, Turgeon RD (2021). Cryoballoon ablation as initial treatment for atrial fibrillation JACC state-of-the-art-review. J Am Coll Cardiol.

[2] Kuck KH, Brugada J, Furnkranz A (2016). Cryoballoon or radiofrequency ablation for paroxysmal atrial fibrillation. N Engl J Med.

[3] Wang Y, Wang W, Yao J, Chen L, Yi S (2021). Second-generation cryoballoon vs contact-force sensing radiofrequency catheter ablation in atrial fibrillation: a meta-analysis of randomized controlled trials. J Interv Card Electrophysiol.

[4] Ravi V, Poudyal A, Pulipati P, Larsen T (2020). A systematic review and meta-analysis comparing second-generation cryoballoon and contact force radiofrequency ablation for initial ablation of paroxysmal and persistent atrial fibrillation. J Cardiovasc Electrophysiol.

[5] Su W, Aryana A, Passman R, Wang P (2018). Cryoballoon best practices II: practical guide to procedural monitoring and dosing during atrial fibrillation ablation from the perspective of experienced users. Heart Rhythm.

[6] Farkowski MM, Karlinski M, Barra S, Boveda S (2022). Effectiveness and safety of a single freeze strategy of cryoballoon ablation of atrial fibrillation: an EHRA systematic review and meta-analysis. Europace.

[7] Holl MJ, Bhagwandien RE, Firouzi M, de Ruiter WA, Szili-Torok T, Yap SC (2021). Reducing radiation exposure in second-generation cryoballoon ablation without compromising clinical outcome. J Interv Card Electrophysiol.

[8] Ciconte G, Sieira-Moret J, Hacioglu E, Chierchia GB (2016). Single 3-minute versus double 4-minute freeze strategy for second-generation cryoballoon ablation: a single-center experience. J Cardiovasc Electrophysiol.

[9] Andrade JG, Champagne J, Dubuc M (2019). Cryoballoon or radiofrequency ablation for atrial fibrillation assessed by continuous monitoring: a randomized clinical trial. Circulation.

[10] Kumar V, Wish M, Venkataraman G, Bliden K, Jindal M, Strickberger A (2019). A randomized comparison of manual pressure versus figure-of-eight suture for hemostasis after cryoballoon ablation for atrial fibrillation. J Cardiovasc Electrophysiol.

[11] Heeger CH, Bohnen JE, Popescu S, Richard Tilz R (2021). Experience and procedural efficacy of pulmonary vein isolation using the fourth and second generation cryoballoon: the shorter, the better?. J Cardiovasc Electrophysiol.

[12] Mugnai G, de Asmundis C, Hunuk B, Chierchia GB (2016). Improved visualisation of real-time recordings during third generation cryoballoon ablation: a comparison between the novel short-tip and the second generation device. J Interv Card Electrophysiol.

[13] Yap SC, Szili-Torok T (2022). Optimization of the transseptal procedural workflow using a novel integrated dilator and needle during a cryoballoon procedure. HeartRhythm Case Rep.

[14] Yap SC, Anic A, Breskovic T, Luik A (2021). Comparison of procedural efficacy and biophysical parameters between two competing cryoballoon technologies for pulmonary vein isolation: insights from an initial multicenter experience. J Cardiovasc Electrophysiol.

[15] Miyazaki S, Kajiyama T, Yamao K, Iesaka Y (2019). Silent cerebral events/lesions after second-generation cryoballoon ablation: how can we reduce the risk of silent strokes?. Heart Rhythm.

[16] Greenstein E, Passman R, Lin AC, Knight BP (2012). Incidence of tissue coring during transseptal catheterization when using electrocautery and a standard transseptal needle. Circ Arrhythm Electrophysiol.

[17] Inohara T, Gilhofer T, Luong C, Tsang M, Saw J (2022). VersaCross radiofrequency system reduces time to left atrial access versus conventional mechanical needle. J Interv Card Electrophysiol.

[18] Demo H, Aranda C, Razminia M. Fluoroless left atrial access for radiofrequency and cryoballoon ablations using a novel radiofrequency transseptal wire. J Interv Card Electrophysiol. 2022;64:183–90. 10.1007/s10840-022-01157-5 PMC9236982 35194727

[19] Stroker E, De Greef Y, Schwagten B, de Asmundis C (2019). Over-the-needle trans-septal access using the cryoballoon delivery sheath and dilator in atrial fibrillation ablation. Pacing Clin Electrophysiol.

